# Charge transport mechanism in networks of armchair graphene nanoribbons

**DOI:** 10.1038/s41598-020-58660-w

**Published:** 2020-02-06

**Authors:** Nils Richter, Zongping Chen, Alexander Tries, Thorsten Prechtl, Akimitsu Narita, Klaus Müllen, Kamal Asadi, Mischa Bonn, Mathias Kläui

**Affiliations:** 1 Johannes Gutenberg-Universität Mainz, Institut für Physik, Staudingerweg 7, 55128 Mainz, Germany; 2 Graduate School of Excellence Materials Science in Mainz, Staudingerweg 9, 55128 Mainz, Germany; 3 Max Planck Institut für Polymerforschung, Ackermannweg 10, 55128 Mainz, Germany; 4 School of Materials Science and Engineering, Zhejiang University, 38 Zheda Road, 310027 Hangzhou, China; 5 Johannes Gutenberg-Universität Mainz, Institut für physikalische Chemie, Duesbergweg 10–14, 55128 Mainz, Germany

**Keywords:** Condensed-matter physics, Materials science

## Abstract

In graphene nanoribbons (GNRs), the lateral confinement of charge carriers opens a band gap, the key feature that enables novel graphene-based electronics. Despite great progress, reliable and reproducible fabrication of single-ribbon field-effect transistors (FETs) is still a challenge, impeding the understanding of the charge transport. Here, we present reproducible fabrication of armchair GNR-FETs based on networks of nanoribbons and analyze the charge transport mechanism using nine-atom wide and, in particular, five-atom-wide GNRs with large conductivity. We show formation of reliable Ohmic contacts and a yield of functional FETs close to unity by lamination of GNRs to electrodes. Modeling the charge transport in the networks reveals that transport is governed by inter-ribbon hopping mediated by nuclear tunneling, with a hopping length comparable to the physical GNR length. Overcoming the challenge of low-yield single-ribbon transistors by the networks and identifying the corresponding charge transport mechanism is a key step forward for functionalization of GNRs.

## Introduction

Two-dimensional materials have drawn immense interest as potential basis for a next generation of electronics with exceptional properties^1^. Various novel low–dimensional materials have recently become available^2^, and among them, graphene nanoribbons (GNRs), one-dimensional stripes of graphene, exhibit numerous exciting physical phenomena: For example, photoluminescence^3^ or non-trivial topological electronic states can arise, where the latter turn GNRs into prototype materials for one-dimensional symmetry-protected topological phases^4,5^. Superlattices of GNRs, e.g. in twisted bilayers or in arrays of GNRs, offer further possibilities to induce and tune exotic quantum states, such as 1D topological superconductivity and Majorana-type states^4,6,7^. In all these cases, ribbon width and their edge structure and functionalization are key parameters determining the electronic structure and, in addition, these parameters also determine the energy gap in the band structure^8–11^. A band gap is not present in graphene and is, however, a prerequisite for many semiconductor applications. The bottom-up synthesis of GNRs provides access to atomically accurate systems, starting from specifically designed molecular precursors, and has therefore attracted much attention^12–16^. Especially the use of the chemical vapor deposition (CVD) method allows for the scalable, cost-effective and high-throughput production of high-quality GNR films^17,18^. The precision of the synthesis together with the versatility of different GNR structures make them ideal objects for testing theoretical predictions concerning their electronic and magnetic properties^8,10,19,20^. Among those GNRs achievable by CVD^12^, ribbons with armchair edges and a width of five carbon atoms^18,21^ (5-AGNRs) are particularly interesting for charge transport because they exhibit a particularly low band gap, as predicted theoretically and confirmed spectroscopically^9–11,22^. Furthermore, photoconductivity measurements on 5-AGNRs indicate very high mobility of charge carriers in these nanoribbons^21,23^. Despite the evident promise of 5-AGNRs, they have not been well studied in devices^18^. And, most importantly, many of the fundamental charge transport mechanisms of GNRs have so far remained unclear. Up to now, most device studies on bottom-up GNRs have aimed at observing charge transport through single ribbons, employing short-channel field-effect transistors (FETs)^24–27^. While these devices show promise for nano-electronics applications, they are typically highly resistive, which has been attributed to large energy barriers at the contacts for charge injection^17,24,26–29^. The recent use of nine-atom-wide AGNRs (9-AGNRs) has enabled substantial improvement regarding conductivity and electrostatic current modulation compared to other previously used GNRs^26^. Still, the challenge of fabricating reliable and reproducible devices based on isolated GNRs, impedes the device characterization such as the extraction of Schottky-barrier heights at the contacts. As alternative to short-channel devices, networks or arrays of GNRs can be used as a basis for a field-effect transistor^30,31^. Such thin film devices have several advantages, including a scalable fabrication process, providing a broad scope of applications, not only as transistors but also, for example, in optoelectronic and chemical sensing. Here, we show that GNR network-based charge transport experiments provide a means to elucidate the physics underlying inter- and intra-ribbon charge transport.

We investigate charge transport in networks of bottom-up synthesized 5-AGNRs and 9-AGNRs grown by CVD. The 5-AGNR serves as a model system to identify the relevant charge transport mechanism in networks, as they are highly conductive with a resistance two orders of magnitude lower than that of 9-AGNR. We have developed a dependable and reproducible device fabrication protocol, optimized for stable and reproducible device features instead of high performance. This fabrication scheme enables reliable transport measurements of the GNR networks and allows a direct comparison of the two ribbon types. In contrast to single-ribbon devices with Schottky-barrier dominated contacts^17,24,26^, the charge injection is not limited in our network FETs with Ohmic contacts, allowing for detailed electrical characterization. Measurements over a previously inaccessible wide temperature range reveal the dominant charge transport mechanism to be nuclear tunneling-assisted carrier hopping. Based on this model, the universal scaling allows us to collapse all the charge current characteristics obtained across several orders of magnitude of bias voltages and temperatures onto a single curve. From this curve, we determine a consistent charge carrier hopping distance.

## Results

### Device fabrication

**Figure 1:**
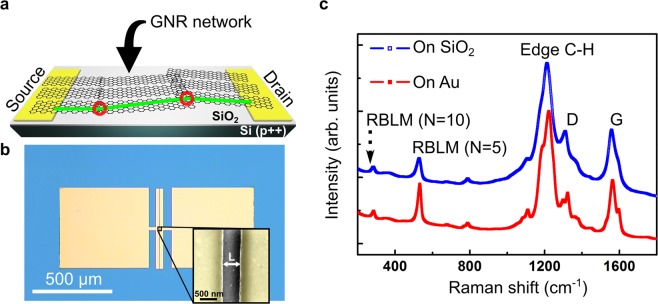
(**a**) Schematic depiction of a GNR network FET where a possible percolation path is drawn in green. The red circles mark locations of charge transfer between the densely packed ribbons. The current flows between the metallic (Au) source and drain electrodes through the GNR channel. The GNR film covers the whole substrate surface. The drawing is not to scale. (**b**) Optical micrograph of a GNR network FET. The SiO_2_ surface appears blue, while the metallic contacts are golden. The inset shows scanning electron microscopy image magnifying the channel region where charge current flows through GNR networks (Au electrodes false-colored in yellow). The junction has a separation of *L* ≈ 600 nm. (**c**) Raman spectrum of a 5-AGNR film before and after the transfer from an Au substrate to a SiO_2_ surface. The spectrum exhibits the usual D (at approximately 1340 cm^−1^) and G (between 1565 cm^−1^ and 1595 cm^−1^) peaks of crystalline sp^2^ carbons. The peak at approximately 1220 cm^−1^ indicates the presence of carbon-hydrogen bonds, located along the periphery of all ribbons. The low-frequency lines can be attributed to the width-dependent RBLMs^21^, where the width is denoted by *N*, the number of carbon atoms across the ribbon.

Figure 1(a,b) present a schematic depiction and an optical micrograph of a typical GNR network FET device. A heavily doped silicon wafer served simultaneously as substrate and back gate electrode. The gate electrode was separated from the lateral GNR channel by a 300 - nm-thick silicon oxide layer. For device fabrication, electron beam lithography was used to define 25 - nm-thick Au source and drain electrodes with a thin Cr layer (5 nm) as an adhesion layer. The channel length was varied from approximately 500 nm to 5 μm, and the channel width was set to a constant value of 500 μm. Finally, the CVD-grown 5-AGNR films and 9-AGNR films, respectively, corresponding to a monolayer of ribbons, were transferred on top of the electrodes. In the case of 9-AGNR two monolayers have been transferred consecutively to obtain sufficient conductance for the variable temperature measurements, especially in the low-temperature range. This technique has been used already in the past successfully for upscaling graphene transistors^32^. To synthesize and transfer the films, our previously reported^17,21^ techniques were employed (for more details on the device fabrication see also the Methods section). The ribbons within the films lie close to one another in a co-planar fashion. As such, they are forming a densely packed ribbon network as revealed in previous studies by scanning tunneling microscopy (STM) of the films as-prepared on the Au growth-substrate^21,33^. Hence, the topography allows for inter-GNR charge carrier transfer, and therefore macroscopic charge currents can be established via percolation paths. As demonstrated for example in refs. ^13,15,27^, Raman spectroscopy can be employed to confirm the integrity of the transferred GNR networks. In Fig. 1(c), we show the Raman spectra of GNR networks transferred on top of our device structures. The Raman spectra are similar with identical peak positions before and after the transfer (Fig. 1(c) for 5-AGNRs and Fig. S1 in the Supplementary Information for 9-AGNRs). Moreover, the width of the GNRs constituting the films can be unambiguously confirmed by the Raman response associated with the radial breathing-like mode (RBLM)^21,34^ showing that the GNRs are not significantly impacted by the transfer.

The intense RBLM peak at 533 cm^−1^ can be assigned to 5-AGNRs. Additionally, a small RBLM peak is visible at 283 cm^−1^, indicating the presence of 10-AGNRs with double the width of 5-AGNRs. A small fraction of 10-AGNRs is formed by a lateral fusion of two 5-AGNRs at the annealing temperature of 400 °C, as has been previously reported by us^21^. One can, however, neglect the contribution of the small number of 10-AGNRs to the charge transport experiments as they have a larger band gap than 5-AGNRs^21^.

### Electrical characterization of the network

**Figure 2:**
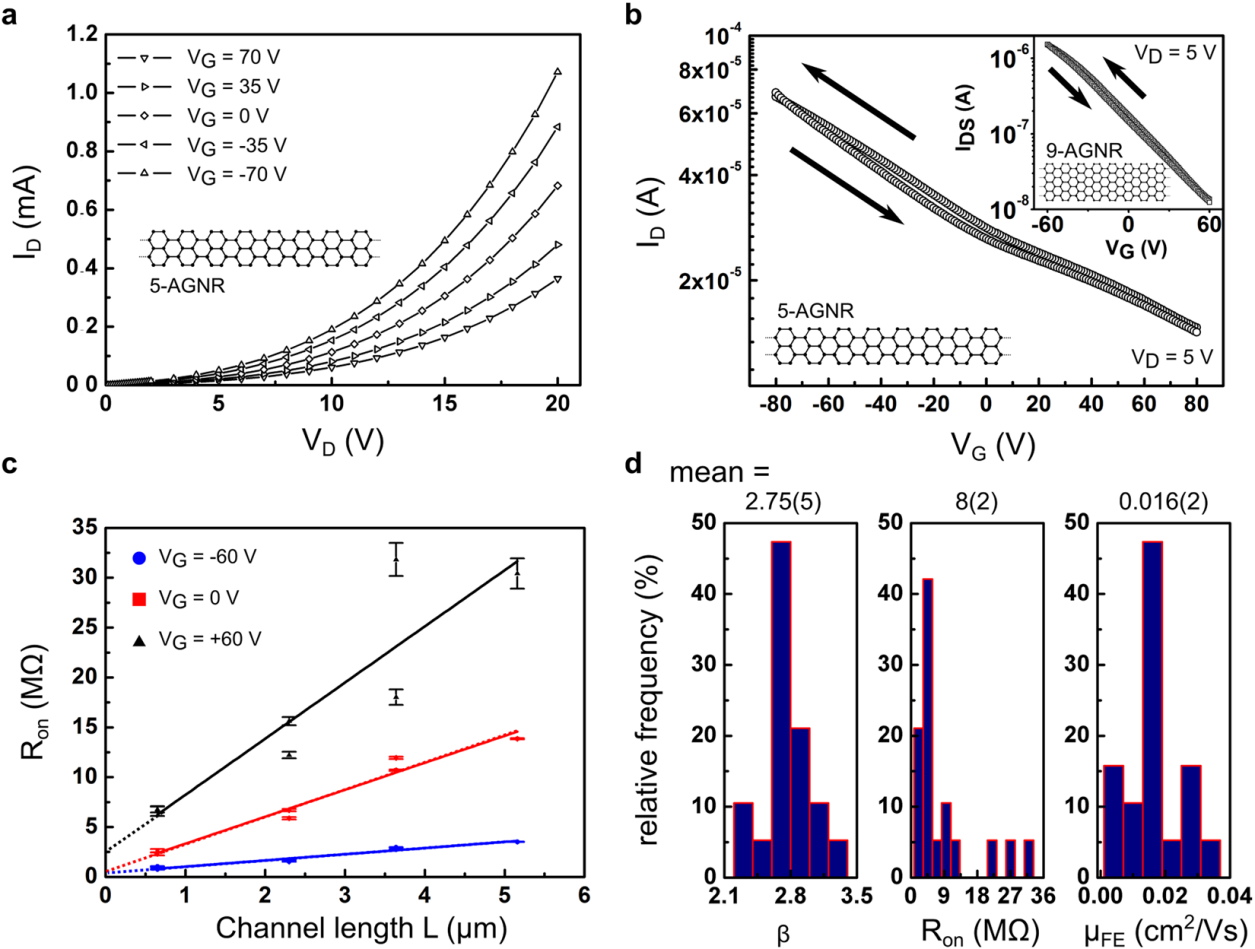
Plot of output (**a**) and transfer curves (**b**) of 5-AGNR. The channel current *I*_*D*_ responds in an Ohmic–like fashion at low *V*_*D*_ (*V*_*D*_ ≤ 1*V*). In (**b**), the arrows indicate the sweep direction and the inset shows a transfer curve measured for a 9-AGNR network device, which is roughly two orders of magnitude less conductive. Lines in (**a**) are guides for the eye. (**c**) Total device resistance (channel and contact resistance) *R*_on_ as a function of channel length for 5-AGNR. Solid lines are linear fits to the data and the dotted lines show the extrapolation to zero channel length, indicating contact resistance. (**d**) Device parameter spread at room temperature. Relative frequency of the values for the exponent *β*, the Ohmic resistance *R*_on_ and the mobility, measured for 19 5-AGNR devices with the same channel length. Mean values and statistical errors of the last digit of the mean values (in parentheses) are indicated above the histograms.

At room temperature and in an inert gas atmosphere, we measured the FET output characteristics of the 5-AGNR network by fixing *V*_*G*_, the voltage applied to the gate electrode, and sweeping the drain voltage *V*_*D*_ from 100 mV to 20*V* and back, with a grounded source electrode. The drain current *I*_*D*_ was measured, with parasitic leakage currents through the gate oxide negligible compared to *I*_*D*_ as shown in Fig. S2 of the Supplementary Information. To avoid artifacts, we ensured the drain current to be always much larger than the gate leakage current. Representative output curves at five different gate voltages from +70*V* to −70*V* are presented in Fig. 2(a). Clearly, different gate biases modulate the conductance of the network. The current increases linearly with increasing drain voltage for *V*_*D*_ < 1*V*, where we deduce the Ohmic resistance as the reciprocal slope *R*_on_. The current follows a power law *I*_*D*_ ∝ *V*_*D*_^*β*^,*β* > 1 for larger voltages (see further modelling below). Hysteresis in the output curves was negligible for all measurements. The transfer characteristics of the GNR-network FET are shown in Fig. 2(b). The device showed minor hysteresis between forward and backward sweeps at the tested sweeping speeds ranging between ~4 V/s and ~100 V/s. Since a saturation region is not reached in the transfer curves, the on/off-ratio *I*_on/off_, which is often used to judge device performance, cannot be determined for our devices. Nevertheless, we can calculate a current modulation ratio *I*_*VG*,1/*VG*,2_ and the field-effect mobility *μ*_*FE*_ based on the obtained data (see S3 in the Supplementary Information for more details on these definitions). Here, these parameters amount to *I*_−80*V*/+80*V*_ ≈ 5 and *μ*_*FE*_ ≈ 2 × 10^−2^ cm^2^ *V*^−1^*s*^−1^, respectively. The current modulation ratio of the 5-AGNR network is low due to the presence of a high background of charge carriers. Using Ohm’s law, this background density can be directly estimated *n*_0_ = *eI*_*D*_*μ*_*FE*_/*V*_*D*_ = 2 × 10^12^ cm^−2^ (at *T* = 260*K*), which is comparable to the electrostatically induced charge carrier density *n*_ind_ = *C*_Ox_*V*_*G*_/*e* ≈ 3.6 × 10^12^ cm^−2^(at *V*_*G*_ = 50V). Since the expected band gap of ~1.7 eV^10^ is much larger than the thermal energy *k*_*B*_*T* at room temperature, the large charge carrier density can be attributed to extrinsic doping (see also S5 of the Supplementary Information).

The transfer characteristics of the 9-AGNR network FETs are shown in the inset of Fig. 2(b). For the shown device, we find *μ*_*FE*_ ≈ 1 × 10^−3^ cm^2^ *V*^−1^*s*^−1^, and a current modulation of *I*_−60*V*/+60*V*_ ≈ 120, the latter is comparable to the reported short-channel devices of 9-AGNR with thick SiO_2_ gate barriers^26^.

Formation of low resistance Ohmic contacts between the GNR network and the FET electrodes is crucial for the reliable extraction of the transport properties^35^. Hence, we analyzed the channel length scaling of the output and transfer curves to retrieve information on the Au/5-AGNR junction. To this end, the transmission line method (TLM)^36^ was applied. A typical TLM plot is shown in Fig. 2(c) for three different gate voltages (−60*V*, 0*V* and +60*V*) for 5-AGNR FETs. TLM is applicable for the whole range of applied gate voltages and the normalized contact resistance *R*_*C*_*W* varies between ~210 Ωm at *V*_*G*_ = −60V and ~320 Ωm for *V*_*G*_ = 0 and higher. We note, that *R*_*C*_ showed a slight dependence on the gate bias as shown in S4 of the Supplementary Information similar to carbon nanotubes^37^. Furthermore, at zero gate voltage, the ratio of the contact resistance versus the total channel resistance (for *L* = 1 *μm*) is < 0.2. Therefore, the channel conductance is dominated by the conductance of the 5-AGNR network and not by the sporadic contact resistance. As a fundamental difference between 5-AGNR and 9-AGNR in the contact resistance formation is not expected, the following analyses are encouraged.

The FET fabrication process is robust with a high device yield approaching 100%. This is evidenced by a comparison of tens of devices on different chips for both types of GNRs (in total 43 devices for 5-AGNRs and 20 devices for 9-AGNRs). Figure 2(d) displays histograms of the relative frequency for three important transport quantities for all 5-AGNR devices, which have the same channel length: The exponent *β*, the Ohmic resistance *R*_on_ and the mobility *μ*_*FE*_. The narrow distribution for each of these device parameters highlights the high homogeneity and uniformity of the GNR networks, and device reproducibility. Especially, the low error for the mean value of *β* of only 2% is noteworthy, since *β* is directly connected with the underlying charge transport mechanism (as detailed below). Clearly, the mobility is not as high as in some large flakes of (gapped) 2D semiconductors such as MoS_2_ or WSe_2_ where values in the range of 10^2^−10^3^ cm^2^*V*^−1^*s*^−1^ are commonly found^1^. However, here we focus on reproducible properties that are found for network devices that currently exhibit reduced mobilities. Yet, further device optimization bears the potential to enhance the mobility by orders of magnitude. For example, choosing suitable substrates is a known path in graphene research, where encapsulation in hexagonal boron nitride enables record mobilities, too^38,39^.

### Universal scaling of charge transport in graphene nanoribbon networks

**Figure 3:**
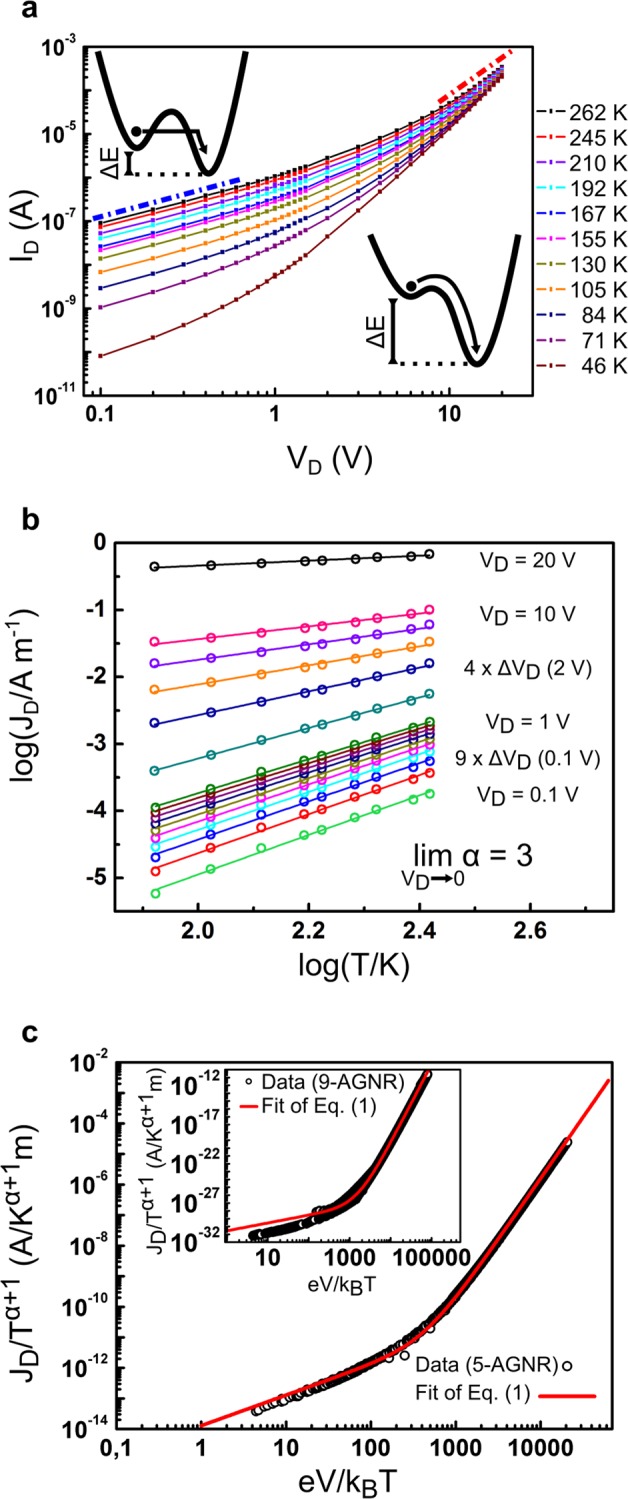
Temperature dependence of charge transport in 5-AGNR networks. (**a**) Shows the evolution of output curves with temperature. Solid lines are guides for the eye. The dashed lines indicate the linear low bias regime (blue) and non-linear high bias regime (red). The charge transport mechanism for low and high bias is shown schematically. In (**b**), we show cuts through the output curves for fixed drain voltages as indicated in the figure. For this plot, the current has been converted to a current density *J*_*D*_ = *I*_*D*_/*W*. Here, lines represent linear fits through the data to determine the exponent of a power law *I*_*D*_ ∝ *T*^*α*^. In (**c**) we plot the scaled channel current density *J*_*D*_/*T*^*α*+1^ as a function of relative energy *eV*/*k*_*B*_*T*. The solid red line is a fit of Eq. 1 with excellent agreement with the measurement. The inset shows the universal scaling curve for 9-AGNRs.

Having established the reproducibility of 5-AGNR network devices, we proceed to investigate the charge transport mechanism. The precise mechanism of charge transport is not a priori evident since the channel length is much longer than the length of individual nanoribbons. Hence, charge carriers must cross ribbon–ribbon junctions in the GNR networks to allow for macroscopic current, suggesting that inter-GNR hopping will contribute to the overall transport. The temperature dependence of charge transport can help to identify the nature of the charge transport mechanism in the network. Therefore, a helium bath cryostat was used to measure the output characteristics from ~262*K* down to ~5K (*V*_*G*_ = 0*V*) for a device with channel length *L* = 1 μm (Fig. 3(a)). At low voltages, the transport is Ohmic, while at higher voltages, the current grows superlinearly with *V*_*D*_, following a power law *I*_*D*_ ∝ *V*_*D*_^*β*^, with *β* = 2.76 ± 0.04 at *T* = 262*K* and zero gate voltage, where gate modulation leaves these functional dependencies unchanged. Such behavior indicates that the charge transport mechanism is through inter-ribbon hopping^40,41^. To describe the charge transport, we employ a quantum mechanical model of dissipative tunneling in a biased double well, mediated by nuclear vibrations, which act as a heat bath^42,43^. In this so-called nuclear tunneling mechanism, the coupling of the electronic charges to their nuclear environment defines the potential energy landscape for charge motion and this motivates the term “nuclear” tunneling. Intuitively, in the low bias regime, charge transport occurs predominantly by tunneling between two electronic states through the energy barrier, and it is temperature dependent because of the coupling of the charge to the nuclear environment. The energy difference between two states depends on the applied electric field V/L and the distance between the initial and the final state L_ij_: Δ*E* = *eL*_*ij*_*V*_*D*_/*L*. At sufficiently high bias however, the double well becomes so asymmetric that the charge carrier can overcome the energy barrier at no energy costs, and therefore the transport becomes virtually temperature independent.

The hopping rate equation and the resulting current have been derived by Fisher and Dorsey, and Grabert and Weiss^42,43^, to read

1 $${I}_{D}={I}_{0}{T}^{1+\alpha }\,\sinh \left(\frac{\gamma eV}{2{k}_{B}T}\right){\left|\varGamma \left(1+\frac{\alpha }{2}+i\frac{\gamma eV}{2\pi {k}_{B}T}\right)\right|}^{2},$$

where *γ*^−1^ is the number of hops that a charge experiences when travelling from one electrode to the other, *α* is a scaled version of Kondo parameter describing the coupling between the charges and the heat bath, and Γ represents the complex gamma function. In the limit $\mathop{\mathrm{lim}}\limits_{V\to 0}$, the current is Ohmic and given by:

2 $$\mathop{\mathrm{lim}}\limits_{V\to 0}{I}_{D}=\frac{{I}_{0}\gamma e}{2{k}_{B}T}{\left|\varGamma \left(1+\frac{\alpha }{2}\right)\right|}^{2}{T}^{\alpha }V,$$

For $\mathop{\mathrm{lim}}\limits_{V\to \infty }$, the current is temperature independent, and has a power law dependence on voltage:

3 $$\mathop{\mathrm{lim}}\limits_{V\to \infty }{I}_{D}={I}_{0}{\pi }^{-\alpha }{\left(\frac{\gamma e}{2{k}_{B}}\right)}^{\beta }{V}^{\beta },$$

The exponents *α* and *β* have to follow the relation *β* = *α* + 1. We determined the exponent *α* from the temperature dependent linear part of the curves using Eq. 2: Log(*J*_*D*_) as a function of log(*T*) at different drain voltages is plotted in Fig. 3(b), where *J*_*D*_ = *I*_*D*_/*W*. The slopes of linear models for each *V*_*D*_ are obtained by a least squares fit giving in the limit of vanishing drain voltage $\mathop{\mathrm{lim}}\limits_{V\to 0}\alpha =3.0\pm 0.1$. At high bias and low temperature, we experimentally find that the exponent *β* in Eq. (3) becomes *β* ≈ 4, validating the prediction *β* = *α* + 1 and corroborating the applicability of this model. Together, these experimental findings suggest that all measurements of *I*_*D*_ at different temperatures and voltages can be combined to a single curve, when *J*_*D*_/*T*^*α*+1^ is plotted as a function of relative energy *eV*/*k*_*B*_*T* as demonstrated in Fig. 3(c). This plot consists of 1552 data points which stem from the output curves between 262K and 46K and from a temperature sweep at a fixed high bias voltage *V*_*D*_ = 10*V* down to 5.6K (see Fig. S5 in the Supplementary Information). By fitting Eq. 1 to the scaled curve with *J*_0_ and *γ* as the only free fitting parameters, we found excellent agreement with *J*_0_ = (8.3 ± 0.2) × 10^−13^A*K*^−α−1^*m*^−1^ and *γ* = (17.6 ± 0.2) × 10^−3^, over the entire range of gate voltages in our experiment.

To check the universal character of this analysis, the temperature dependent charge transport experiments were repeated for an FET based on 9-AGNR networks with a channel length of *L* = 2 μm. Here as well, the universal scaling is applicable with *α* = 9, *J*_0_ = (2.1 ± 0.1) × 10^−32^AK^−α−1^m^−1^ and *γ* = (9.7 ± 0.1) × 10^−3^. Finally, while properties such as the conductivity of 5-AGNR and 9-AGNR vary strongly, the extracted values for *γ* are similar. This dependence of the hopping rate *γ* can serve as an independent check of our conclusion, that inter-GNR hopping limits charge mobility in our network devices. From the model we see that the product *L* × *γ* yields the statistical average of a hopping length. We find for both 5-AGNR and 9-AGNR an average distance between charge carrier hops of 17 to 19 nm. This hopping length is in fact comparable to the average length of individual GNRs in the network^17,21^ showing that the analysis is robust. This geometrical agreement implies that the limiting factor for charge transport in the network is inter-ribbon hopping. The intra-ribbon mobility on the other hand can be orders of magnitude larger, supported by observed band-like transport in the GNRs^13,17,21–23,40,44,45^.

## Conclusion

We have demonstrated reproducible FETs based on networks of 5- and 9-AGNRs. The network-based FETs do not rely on identifying and contacting individual ribbons and are therefore able to boost the fabrication yield. The device properties are reproducible, with a narrow spread in parameters. The FETs based on 5-AGNR showed remarkable high conductance compared to 9-AGNRs with a difference of at least two orders of magnitude. Through a systematic analysis of *I*(*V*,*T*) characteristics, we were able to determine the nature of charge transport characteristics in both GNR networks. We found that the transport characteristics can be described by a universal scaling based on a fully quantum mechanical hopping transport mechanism. For both 5- and 9-AGNRs, the different *I*(*V*,*T*) characteristics could be collapsed onto a single universal curve, indicating the generality of the transport mechanism. The universal curves integrated measurements at temperatures between 5*K* to 262*K* for voltages, swept over two orders of magnitude. The modeling determined the hopping of the charge carriers between nanoribbons as the factor limiting charge transport. The use of long GNRs in networks will therefore enable higher field-effect mobilities for future GNR-based FETs.

## Methods

### Growth of graphene nanoribbons by chemical vapor deposition

All 5-AGNRs and 9-AGNRs were synthesized from surface-assisted chemical vapor deposition (CVD) technique as we reported previously elsewhere^17,21,33^. The CVD system comprises a horizontal tube furnace (Nabertherm, RT 80-250/11 S) and heating belt (Thermocoax Isopad S20). The Au/mica substrate was loaded into the tube furnace as the growth substrates and heated to 250 °C under a gas flow of Ar (500 sccm) and H_2_ (100 sccm) with a pressure of ~1.5 mbar. The precursor for 5-AGNRs, an isomeric mixture of 3,9-dibromoperylene and 3,10-dibromoperylene (DBP), was then sublimed by the heating belt at ~250 °C and deposited on the Au/mica substrate for 30 min for polymerization and subsequently annealed at 400 °C for 15 min for cyclodehydrogenation.

Similarly, for the synthesis of 9-AGNRs, the Au/mica substrates were loaded into the tube furnace and heated to 200 °C under a gas flow of Ar (500 sccm) and H_2_ (100 sccm) with a pressure of ~1.5 mbar. At the meantime the monomer 3′,6′-dibromo-1,1′:2′,1″-terphenyl was loaded upstream and sublimed at 150 °C for 30 min for polymerization. Subsequently, the samples were annealed at 400 °C for 15 min for cyclodehydrogenation.

### Device fabrication

The heavily doped silicon wafers, which served as both substrate and back gate electrode, are commercially available and have a 300 nm thick silicon oxide layer (thermally oxidized). The wafers have been diced into chips of 1 × 1 cm^2^. We used electron beam lithography to define 25 nm thick Au source and drain electrodes where a thin layer Cr (5 nm) served as an adhesion layer. Finally, GNR films were transferred on top of the structures. In the case of 9-AGNRs, we transferred two layers.

### Transfer of GNR thin films

The procedure to transfer the films, we employed a technique which we have reported previously^17^: After the CVD growth of the GNRs, a thin layer of poly(methyl methacrylate) (PMMA) was spun onto the GNR/gold/mica stack which provided additional mechanical stability and facilitated the transfer of intact films over a large area. Carefully, the resulting stack was floated on concentrated HF for several hours to delaminate the PMMA/GNR/gold film from the mica slab. After the delamination was complete, the gold was etched away in a gold etchant (Sigma-Aldrich). We then transferred the PMMA/GNR film to the target substrate with Au electrodes. To dissolve the PMMA, the PMMA/GNR/substrate stack was immersed in an acetone bath. Finally, we rinsed the chip with isopropanol and dry blow the GNR film.

### Raman spectroscopy

Raman characterization of the GNR films was performed with a Bruker SENTERRA RFS100/S Raman spectrometer using a 785 nm laser under ambient conditions.

### Room temperature electrical characterization

Measurements have been performed using a three-terminal probe station integrated into an inert gas glove box with a nitrogen atmosphere. The probe station was connected to a Keithley 4200 semiconductor characterization system, which contains three independent source-measure units and allows for the electrical characterization of test devices.

### Variable temperature electrical characterization

Variable temperature measurements have been carried out in a bath cryostat equipped with a dynamic variable temperature insert (VTI) at the sample space, which allows for the control of the sample temperature. Stable temperatures within approximately 0.1*K* are obtained by balancing the cooling power of a liquid helium flow against the heating power of an electrically resistive heating element. The liquid helium is drawn from the main reservoir through a needle valve, which is adjusted manually. Temperatures below 4.2K are obtained by reducing the vapor pressure of the liquid helium in the sample space by mechanical pumping. Electrical measurements are enabled with a Keithley 238 source-measure combined with a Keithley 2400 source-measure unit. To accurately measure high resistive samples in this setup, we employed a current guarding method.

## Supplementary information

Supplementary Information.
