# Supplementary material for: Charge transport mechanism in networks of armchair graphene nanoribbons
Source: Sci Rep. 2020 Feb 6;10:1988. doi: 10.1038/s41598-020-58660-w (PMC7005326; doi:10.1038/s41598-020-58660-w)
Supplement: Supplementary file 1 — Supplementary Information. [file 41598_2020_58660_MOESM1_ESM.pdf]

Supplementary Information

Charge transport mechanism in networks of armchair graphene nanoribbons

Nils Richter[1,2], *Zongping Chen*[3,4], *Alexander Tries*[1,2,3], *Thorsten Prechtl*[3,5], *Akimitsu Narita*[3], *Klaus Müllen*[2,3,5,*], *Kamal Asadi*[3], *Mischa Bonn*[2,3], *Mathias Kläui*[1,2,*]

[1]Johannes Gutenberg-Universität Mainz, Institut für Physik, Staudingerweg 7, 55128 Mainz, Germany
[2]Graduate School of Excellence Materials Science in Mainz, Staudingerweg 9, 55128 Mainz, Germany
[3]Max Planck Institut für Polymerforschung, Ackermannweg 10, 55128 Mainz, Germany
[4]School of Materials Science and Engineering, Zhejiang University, 38 Zheda Road, 310027 Hangzhou, China
[5]Johannes Gutenberg-Universität Mainz, Institut für physikalische Chemie, Duesbergweg 10–14, 55128 Mainz, Germany

*E-mail: muellen@mpip-mainz.mpg.de
*E-mail: klaeui@uni-mainz.de

S1. Raman spectroscopy of 9-AGNR films

In **Fig. S1** we show Raman spectra taken on a 9-AGNR film as-grown on Au and after the transfer on SiO_2 demonstrating the intactness of the film after the transfer. We find the RBLM peak at approximately 311 cm^{-1}, which is the expected value for 9-atom wide GNRs[1]. As no additional peaks appear we can conclude that the GNRs are not significantly altered by the transfer process.

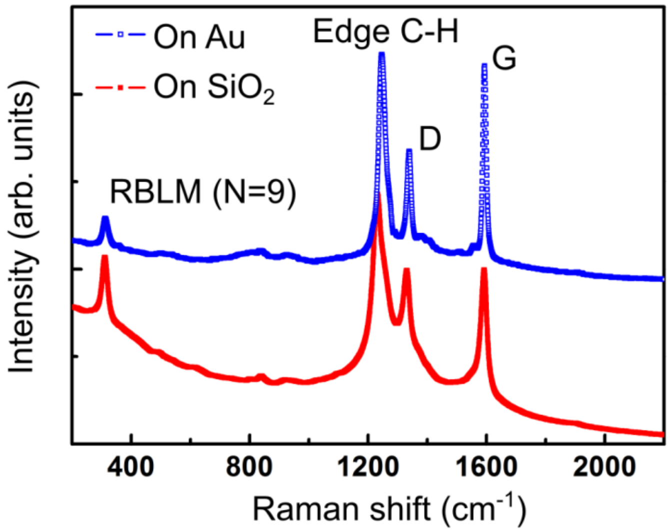

Figure S1. Raman spectrum of a 9-AGNR film on Au and SiO_2 surfaces. The low-frequency line at 311 cm^{-1} can be attributed to the presence of intact 9-AGNRs (the ribbon width is denoted by N, the number of carbon atoms across the ribbon).

S2. Gate-leakage and bias symmetry in 5-AGNR network FETs

As described in the main text, in FET devices parasitic leakage currents can occur through the gate barrier. At high gate voltages and low drain voltages, the leakage leads to small systematic errors in the measurement. In order to quantify the impact of leakage currents, we measure the current at the gate electrode $I_G = I_{DG} + I_{SG}$, where I_{SG} is the current flowing between the source electrode and the gate electrode. As shown in **Fig. S2**, the gate current is small compared to the drain current and its dependence on V_D is negligible at small voltages applied to the gate electrode. Only at large V_G, the leakage starts to affect I_D slightly. However, since I_G does not change with V_D, we correct for its influence by simply subtracting I_G from the I-V curves, such that $\lim_{V_D \to 0} I_D = 0$.

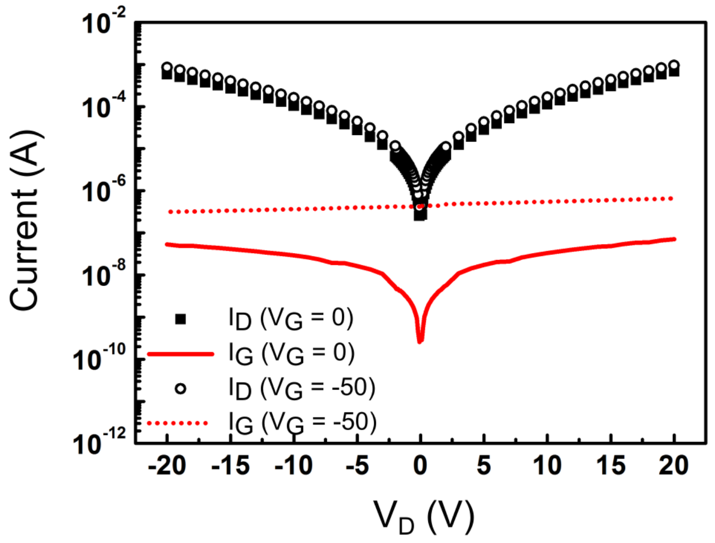

Figure S2. Gate leakage current and bias symmetric channel current for a representative 5-AGNR device presented in a semi-logarithmic plot. The gate current is orders of magnitude below the channel current and does not depend on bias voltage. We plot the absolute value of the channel current in order to provide a facile comparison of positive and negative bias. Although the current at negative drain voltages is systematically lower than the current at positive drain voltages, this difference is very small. Hence, the use of data from only positive bias is justified.

S3. GNR-network FET characterization

In a conventional FET, the ratio $I_{\mathrm{on/off}}$, which compares the current in the saturation regime (on-state) with the current in the subthreshold regime (off-state), is widely used as a figure of merit to quantify the effect of the gate voltage. Since a current saturation regime is not reached in our devices, we define in analogy to $I_{\mathrm{on/off}}$ a current modulation ratio

$$I_{VG,1/VG,2} = \frac{I(V_{G,1})}{I(V_{G,2})}, \tag{S1}$$

where we compute the ratio of channel current at specific gate voltages. Furthermore, the field-effect mobility can be extracted from the transfer curve via[2]

$$\mu_{FE} = \frac{g\,L}{V_D\,C_{0\mathrm{x}}\,W}, \tag{S2}$$

where $g = \partial I_{SD}/\partial V_G$ is the transconductance, L is the channel length, W is the channel width and $C_{0\mathrm{x}} = \epsilon\epsilon_0/t_{0\mathrm{x}} \approx 1.15 \times 10^{-4}\mathrm{F/m^2}$ is the geometrical capacitance density assuming that the channel and the gate electrode form a parallel plate capacitor. Here, we use $t_{0\mathrm{x}} = 300\ \mathrm{nm}$, a relative permittivity of SiO_2 $\epsilon = 3.9$ and a vacuum permittivity $\epsilon_0 = 8.854\ \mathrm{F/m}$. Ideally, the transconductance is determined in the linear regime of the transfer curve. However, with our GNR network FETs, a linear regime is not reached. Therefore, we use a linear approximation of the curve in the range $V_G \leq -35\ \mathrm{V}$. In the linear regime of transfer curves, the channel current is usually much larger than in the subthreshold regime and therefore the transconductance, which we extract in this way leads to a systematic underestimation of μ_{FE}. Hence, the values given for μ_{FE} are lower bounds. However, the contact resistance-free mobilities are of the same magnitude showing that the systematic error in the field-effect mobility is small and hence these values represent a good approximation of the charge carrier mobility in the devices.

With nuclear tunneling-assisted hopping as the dominant charge transport mechanism, we can further rationalize the values for the field-effect mobility. Although a degradation of the charge carrier mobility with larger band gaps is expected, using terahertz spectroscopy, values

for the mobility in the order of 10^2 $cm^2V^{-1}s^{-1}$ have been experimentally observed in 9-AGNR samples[1]. Hence, the mobility of individual GNRs can be much larger than the field effect mobilities determined in our charge transport experiments. On the other hand, when hopping is the dominant charge transport mechanism, mobilities in the range of 10^{-1} $cm^2V^{-1}s^{-1}$ to 10^{-4} $cm^2V^{-1}s^{-1}$ are typically observed[3].

S4. Gate voltage-dependence of the contact resistance

The gate voltage dependent contact resistances were determined for a series of 5-AGNR devices by measuring charge transport at room temperature for various channel lengths and using the transmission line method[3]. The total device resistance consists of the channel resistance and the contact resistance, $R_{\text{on}} = R_{\text{channel}} + R_C$. The channel resistance is a function of gate voltage and is proportional to the geometrical aspect ratio of the channel L/W, with L the channel length and W, the channel width. We use the Ohmic part of the I-V curves at low drain voltages to determine the device resistance R_{on} as the slope of a linear model via a least squares fit. The data points for each gate voltage were again linearly fitted (least squares fit) to extract the total contact resistance (source + drain) R_C from the intercept of the fit curve with the ordinate-axis at zero channel length. In **Fig. 2 (d)** of the main text, we show the result of this procedure for one device at three different gate voltages. The contact resistance R_C is expected to be a constant with respect to the gate voltage. However, due to current crowding effects a gate voltage dependence can be induced[4] and we observe a decrease of R_C when the gate voltage is lowered from large positive values towards zero. When the gate voltage is further lowered towards large negative values, we gradually turn on the conducting channel and the contact resistance becomes constant. This behavior is typical for example in CNT network field-effect transistors[4]. At zero gate voltage, the ratio of contact resistance versus total device resistance (for $L = 1\ \mu m$) is < 0.2. Therefore, we conclude that the contact resistance does not play a dominant role our measurements and we can neglect it in our analysis.

To corroborate this further, we exemplify a correction for the charge carrier mobility and compare the corrected value to the as-measured data. With the help of the width–normalized inverse channel resistance $m = (\partial R/\partial L)^{-1}/W$ at different gate voltages (**Fig. S3**) the contact resistance-corrected charge carrier mobility[3] $\mu_{RC} = 1/C_{0\text{x}}\,(\partial m/\partial V_G)^{-1} = (3.4 \pm 0.0.2) \times$

10^{-3} cm^{2}V^{-1}s^{-1} can be determined. Following this method, any apparent channel-length dependence of the field-effect mobility originating from contact resistance at the source and drain electrodes is eliminated. However, for this set of devices μ_{FE} extracted from the transfer curves ranges from 0.01 cm^{2}V^{-1}s^{-1} to 0.03 cm^{2}V^{-1}s^{-1} ($V_{SD} = 15$ V) showing that the difference is small and thus corroborating that the influence of the contacts is negligible.

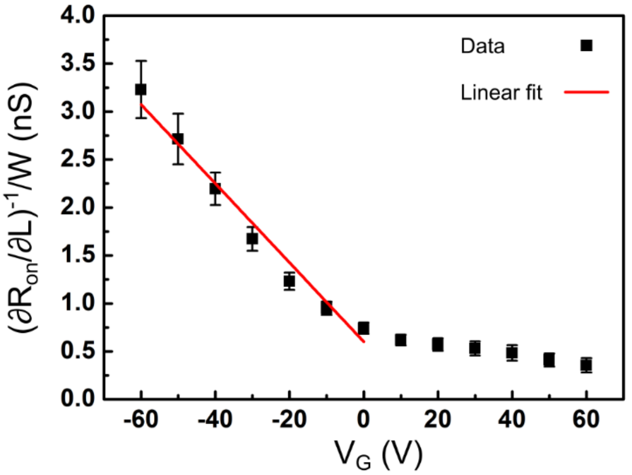

Figure S3: Contact resistance of metal/GNR interfaces. Width–normalized reciprocal slopes of the total resistance depending on gate voltage allowing for the determination of a contact resistance–corrected charge carrier mobility.

S5. Charge carrier density as a function of temperature

For the determination of the charge carrier density as

$$n_0 = \frac{eI\mu}{V} = \frac{e\mu}{R}, \quad \text{(S3)}$$

we combine I-V curves (**Fig. 3 (a)** in the main text) and transfer curves (**Fig. S4**) at variable temperatures. Here, we use the Ohmic part of the I-V curves to determine R and we use μ_{FE} (measured at $V_D = 1$ V) as the mobility. At 260 K, the charge carrier density is high with approximately 2×10^{12} cm^{-2}. This justifies the use of **Eq. 1** of the main text, which requires equal hopping steps through the device channel. Furthermore, the charge carrier density remains constant from 260 K down to approximately 100 K. Only at lower temperatures, the carrier density starts to decrease. Measuring the mobility at constant drain voltage is impeded at lower temperatures due to the large resistance.

The systematic error in the mobility, which we discuss above (**S3**), of course propagates to the charge carrier density. Nevertheless, the temperature dependence of the transfer curves is captured correctly in the field-effect mobility and therefore our results are robust against these systematic uncertainties.

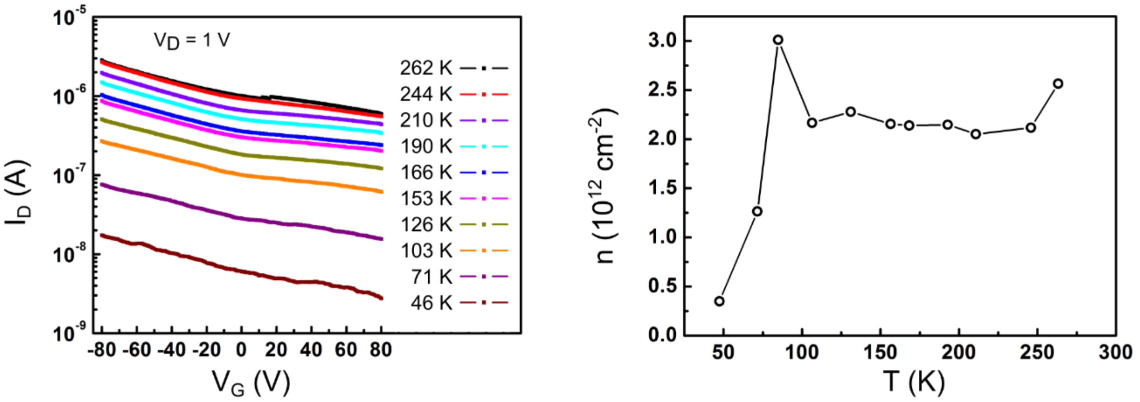

Figure S4. Temperature dependence of charge carrier density 5-AGNR network FETs. In (a), we present the temperature evolution of the transfer curves. The field-effect mobility is extracted from these curves and used to estimate the charge carrier density. As shown in (b), the charge carrier density is constant over a wide temperature range between 100 K and 260 K. Lines in are guides for the eye.

S6. Additional temperature dependent measurements

Additional to the charge transport data from the I-V curves shown in the main text, we performed a temperature sweep down to 5.6 K at fixed $V_D = 10$ V and $V_G = 0$ as shown in **Fig. S5**. These data are also included in the universal scaling shown in **Fig. 3 (c)** of the main text. We also tested our data against a variable range hopping (VRH) model as this is a competing hopping mechanism. However, the model failed to describe the experimental observation thus ruling out this model.

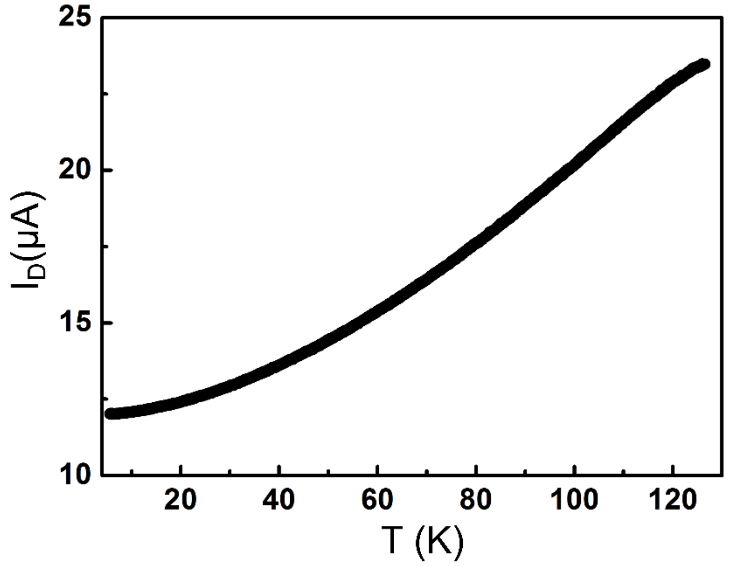

Figure S5. Temperature dependence of the channel current at $V_D = 10$ V and $V_G = 0$.

References

1. Chen, Z. et al. Chemical Vapor Deposition Synthesis and Terahertz Photoconductivity of Low-Band-Gap N = 9 Armchair Graphene Nanoribbons. *J. Am. Chem. Soc.* **139,** 3635–3638 (2017).

2. Choi, H. H., Cho, K., Frisbie, C. D., Sirringhaus, H. & Podzorov, V. Critical assessment of charge mobility extraction in FETs. *Nat. Mater.* **17,** 2–7 (2017).

3. Natali, D. & Caironi, M. Charge Injection in Solution-Processed Organic Field-Effect Transistors: Physics, Models and Characterization Methods. *Adv. Mater.* **24,** 1357–1387 (2012).

4. Schießl, S. P. et al. Polymer-Sorted Semiconducting Carbon Nanotube Networks for High-Performance Ambipolar Field-Effect Transistors. *ACS Appl. Mater. Interfaces* **7,** 682–689 (2015).
